# Neurotic Pruritus

**Published:** 1890-05-03

**Authors:** 


### NEUROTIC PRURITUS.

Dr. Wertheimer, in a case of a woman suffering from universal cutaneous pruritus of nervous origin, tried salicylate of soda in 15 grain doses thrice daily. After the third dose she enjoyed the first night's undisturbed sleep she had had for a long time, and by the fourth day all itching had entirely ceased. Smaller doses were given for a few days longer, and she has remained since free from any return of the pruritus.

